# Prefrontal and sensorimotor cortices showed opposite patterns of hemodynamic activity changes between eyes-open and eyes-closed conditions in blepharospasm

**DOI:** 10.3389/fnagi.2026.1825810

**Published:** 2026-10-15

**Authors:** Xiao-Feng Huang, Zhen Zhang, Li-Na Tan, Xin-qing Hao, Zhan-Hua Liang, Chen-Hui Pei

**Affiliations:** Department of Neurology, The First Affiliated Hospital of Dalian Medical University, Dalian, China

**Keywords:** amplitude of low-frequency fluctuation, blepharospasm, eyes closed, eyes open, functional near-infrared spectroscopy

## Abstract

**Background:**

Blepharospasm (BSP), characterized by involuntary eyelid closure and difficulty opening the eyes, often results in functional blindness. However, there is a lack of objective and quantitative diagnostic markers, and the differences in hemodynamics between eyes-open (EO) and eyes-closed (EC) conditions are unknown in BSP.

**Objective:**

To address this issue, we utilized functional near-infrared spectroscopy (fNIRS) to measure the oxygenation differences in the prefrontal cortex (PFC) and sensorimotor cortex in response to EO and EC conditions and compared them with those of healthy controls.

**Methods:**

We analyzed data from 96 BSP individuals and 31 healthy controls (HCs) by using the amplitude of low-frequency fluctuations (ALFF) determined by functional near-infrared spectroscopy (derived from fNIRS) to measure the oxygenation differences in the prefrontal cortex (PFC) and sensorimotor cortex in response to EO and EC conditions.

**Results:**

The BSP group and the HC group exhibited opposite patterns of differences between the EC and EO conditions. In the HC group, the ALFF was greater in the EC condition than in the EO condition in the frontopolar area (FPA) and orbitofrontal cortex and lower in the EC condition than in the EO condition in the bilateral sensorimotor cortex. In the BSP group, the ALFF was greater in the EC condition than the EO condition in the bilateral sensorimotor cortex and lower in the EC condition than in the EO condition in the frontopolar area and bilateral dorsolateral prefrontal cortex. Moreover, differences in the EC-EO patterns at channel 44 and channel 3 between the two groups showed promising excellent discrimination potential, pending external validation.

**Conclusion:**

The BSP group and the HCs group showed opposite patterns of EO-EC differences in the prefrontal cortex and sensorimotor cortex. The delta EO-EC difference between the right S2 and the right FPA showed promising has excellent differential diagnostic value for BSP in this discovery cohort and is a candidate and is expected to become an early diagnostic biomarker requiring external validation and neuromodulation therapeutic target.

## 1 Introduction

Blepharospasm (BSP) is a common focal dystonia that manifests with involuntary eyelid closure and difficulty opening the eyes due to disturbed activity in the orbicularis oculi and other periocular muscles (Defazio et al., 2013; Defazio et al., 2017). The peak age of BSP onset occurs in the fifth and sixth decades (Lee et al., 2018; Simpson et al., 2016; Fang et al., 2020). Excessive involuntary eyelid closure and difficulty opening the eyes may lead to severe social and daily life disabilities and even functional blindness. The etiology and pathophysiological mechanisms of BSP have not been fully elucidated, and the most effective treatment is botulinum toxin (BoNT) injection into the hyperactive muscles (Defazio et al., 2017; Simpson et al., 2016). Although the efficacy of treatment can be maintained for approximately 2–3 months in more than half of patients, many patients experience nonsignificant improvement in quality of life (Fang et al., 2020; Defazio et al., 2015). Because the effectiveness of BSP treatment is unsatisfactory, many patients suffer from lifelong chronic disability, unemployment, social evasion, depression and even physical and mental hopelessness. BSP is a relatively rare but disabling disorder. Population-based studies have estimated its prevalence at approximately 30–130 per million individuals (Steeves et al., 2012), with a marked female predominance (female-to-male ratio of approximately 2:1) and a peak onset in the fifth to sixth decade of life, indicating that BSP is strongly age-related (Jinnah et al., 2013; Sun et al., 2018). Its clinical burden extends beyond the motor symptom: psychiatric comorbidities are common, with anxiety and depression reported in approximately one-quarter to one-half of patients, and mild cognitive impairment has also been documented (Defazio et al., 2017; Defazio et al., 2022). In addition, BSP shares important clinical and pathophysiological features with other age-related and neurodegenerative movement disorders: abnormal blinking and sensory gating disturbances often precede the onset of overt spasms (Conte et al., 2017), comorbid adult-onset dystonia occurs at an increased rate, and network-level abnormalities in the prefrontal and sensorimotor cortices resemble those described in Parkinson’s disease and other basal ganglia disorders. These features collectively highlight BSP as a model disorder for studying age-related sensorimotor network dysfunction, and they motivate a brain-function-based rather than a purely symptom-based diagnostic approach.

Although the exact etiology of BSP remains unknown, several interrelated mechanisms have been proposed, including impaired sensorimotor integration, reduced cortical inhibition, abnormal synaptic plasticity, and dysfunction of the basal ganglia gating system, which together fail to suppress abnormal motor activity and lead to compensatory hyperactivation of cortical areas (Perruchoud et al., 2014; Horovitz et al., 2012; Xu et al., 2023). Importantly, these proposed mechanisms predict abnormal regional activity and altered coupling within large-scale sensorimotor, prefrontal, and visual networks-changes that are, in principle, detectable with non-invasive functional neuroimaging even in the absence of structural lesions. Accordingly, functional neuroimaging studies have shown that BSP disturbs brain function through dysfunction of one or more communication nodes within networks (Xu et al., 2023; Huang et al., 2017; Mascia et al., 2020). Resting-state functional imaging studies have shown functional abnormalities in several brain regions, including the sensorimotor cortex, prefrontal cortex, cerebellum, and brainstem (Huang et al., 2017; Mascia et al., 2020; Guo et al., 2020). However, the results of these studies are not consistent. In addition, previous studies of BSP have been based mainly on the eyes-closed (EC) condition, and to our knowledge, very few studies have evaluated the characteristics of brain activity in patients with BSP under the eyes-open (EO) condition. BSP patients generally have no obvious spasm in the resting state under the EC condition. However, brain functions during spasm have not been characterized. At present, the diagnosis of BSP is mainly based on typical spasm symptoms. Due to the lack of objective and effective auxiliary diagnostic methods, the early misdiagnosis rate is as high as 90%, and the average delay in diagnosis is approximately 38 months (Huang et al., 2015; Yi et al., 2022). Given the delayed diagnosis, the high psychiatric comorbidity burden, and the network-level nature of the proposed pathophysiology, the development of a simple and rapid brain-function-based candidate biomarker could aid in the early diagnosis and treatment of BSP and reduce the pain of patients who have been seeking medical treatment for a long time but have not been diagnosed accurately.

Previous brain functional studies have confirmed that functional activity in human brain regions and brain networks dynamically switches between eyes-closed (EC) and eyes-open (EO) states (Zhang and Zang, 2023; Costumero et al., 2020). EO-EC switching may transduce interoceptive and exteroceptive signals rather than just being an incoming portal for visual perception. Therefore, it is important to consider carefully whether to choose the eyes-closed or eyes-open state for resting-state brain functional imaging studies (Barry et al., 2017; Jeong, 2014). Recently, resting-state magnetoencephalography studies have shown that differences between the EC and EO states are helpful in differentiating early Alzheimer’s disease (Ikeda et al., 2020). PET studies have shown that, compared with healthy controls, patients with BSP exhibited no significant abnormalities in the striatum at rest with eyes closed, while striatal metabolism was reduced compared with controls in the eyes-open state (Suzuki et al., 2019). This finding indicates that BSP-related functional abnormalities are unmasked by the EO condition rather than by the EC condition, providing direct precedent that the EC-EO contrast is informative for revealing disease-related brain dysfunction in BSP. Clinically, this is especially relevant for BSP because its cardinal symptom-difficulty opening the eyes with involuntary eyelid closure-manifests only when the eyes are open, whereas patients typically show no overt spasm at rest with eyes closed; the EC-EO contrast therefore corresponds directly to the switch between the symptom-silent and symptom-manifest states. Moreover, previous studies in healthy populations have established that EO and EC are distinct, physiologically separable functional states with reliable differences in regional activity and large-scale network organization (Zhang and Zang, 2023; Costumero et al., 2020; Barry et al., 2017; Jeong, 2014; Ikeda et al., 2020; Suzuki et al., 2019; Zou et al., 2015; Liu et al., 2013; Yuan et al., 2018; Xu et al., 2014; Weng et al., 2020), and EC-EO differences have proved useful for differentiating patients from controls in other disorders such as early Alzheimer’s disease (Ikeda et al., 2020). Therefore, EC and EO status are important considerations in the study of brain function in patients with BSP, and identifying differences in EO-EC status may be important for further understanding the pathophysiological mechanism of BSP and developing candidate biomarkers for differential diagnosis. However, to our knowledge, no studies characterizing the EO-EC variability of BSP patients have been conducted.

fNIRS is a noninvasive and portable neuroimaging technique for assessing brain function that can reliably detect changes in oxyhemoglobin in brain tissue and has recently been increasingly used in the study of brain function (Ferrari and Quaresima, 2012). Compared with functional MRI, fNIRS has the advantage of better resistance to motion interference and can enable rapid examination of brain function in subjects in a variety of natural conditions (Pinti et al., 2020). BSP patients are mostly elderly and often have difficulty keeping their head still during resting-state examinations and performing tasks; because fNIRS does not limit occasional postural movements of patients, it may be more suitable for studying brain function in BSP patients (Huang et al., 2023; Butters et al., 2023).

In this study, we used fNIRS to compare the difference in brain functional activity between BSP patients and healthy subjects under EC and EO conditions to investigate the pathophysiological mechanism of BSP and to assess the potential value of the difference in brain activity between EC and EO conditions in distinguishing between BSP patients and healthy subjects.

## 2 Materials and methods

### 2.1 Subjects

A total of 96 BSP patients were included according to the following criteria: (1) diagnosis of primary blepharospasm by two movement disorder experts according to the diagnostic criteria established by the 2020 Chinese Expert Consensus on the Diagnosis of Dystonia and the 2013 International Blepharospasm Working Group (Defazio et al., 2013; Chinese Society of Neurology Chinese Society of Parkinson’s Disease and Movement Disorders, 2020); (2) no abnormal structural lesions on brain imaging; (3) no other neurological diseases or mental illness; (4) right-handed; and (5) at least 3 months since the last botulinum toxin injection. The exclusion criteria were as follows: (1) age ≥ 75 years; (2) other forms of dystonia in other body parts; (3) traumatic brain injury, dementia, essential tremor, other neurological disorders or mental disorders; (4) use of psychotropic or antidopaminergic drugs that may induce blepharospasm; (5) family history of dyskinesia; and (6) symptoms too severe to cooperate with the study. A total of 31 healthy controls were recruited from the community.

### 2.2 Demographic and clinical data

We collected patient’s demographic and clinical data, including age, sex, years of education, disease duration, medical history, total number of injections, and duration of botulinum toxin treatment efficacy. Motor symptom severity was assessed using the Jankovic Rating Scale (JRS) in the patient group (Jankovic and Orman, 1987).

### 2.3 fNIRS data acquisition

We used a multichannel near-infrared brain function imaging device (NirSmart, Danyang Huichuang Medical Equipment Co., Ltd., China) to record cortical neural activity during the resting state. The emission light wavelengths were 730 nm and 850 nm. There were 23 emitters and 15 detectors used to form 47 effective channels. The distance between channels was 3.0 cm, and the sampling rate of all channels was 11 Hz. The probe set was placed on the scalp above the bilateral prefrontal cortex, parietal lobe and temporal lobe (Figure 1). NirSpace (Danyang Huichuang, China) was used as a three-dimensional positioning system to obtain the Montreal Neurological Institute (MNI) coordinates of the channels. During the fNIRS assessment, the participants were seated in a warm, quiet and slightly dark room. The fNIRS data were collected for 5 min in both the eyes-closed and eyes-open states, and the subjects were instructed to relax physically, sit in a comfortable position on the seat, and avoid actively thinking about anything. In the eyes-open condition, the subjects were instructed to maintain central fixation on a black cross-shaped marker (20 cm × 20 cm) affixed to a white board positioned directly in front of them at eye level, at a viewing distance of 1 meter, and to keep their gaze on the marker throughout the 5-min acquisition; they were also instructed not to deliberately control their eyelid movements. The room lighting was held constant across participants and conditions. The face of each participant was video-recorded throughout the acquisition, and subjects with a cumulative eye-closure time exceeding 30 s in the eyes-open condition were excluded from the analysis. All included participants completed the full 5-min EO acquisition and remained below the exclusion threshold. Although cumulative eye-closure time was systematically reviewed for exclusion purposes, episode-level counts of ocular spasms were not prospectively quantified; we acknowledge this as a limitation of the current study.

**Figure 1:**
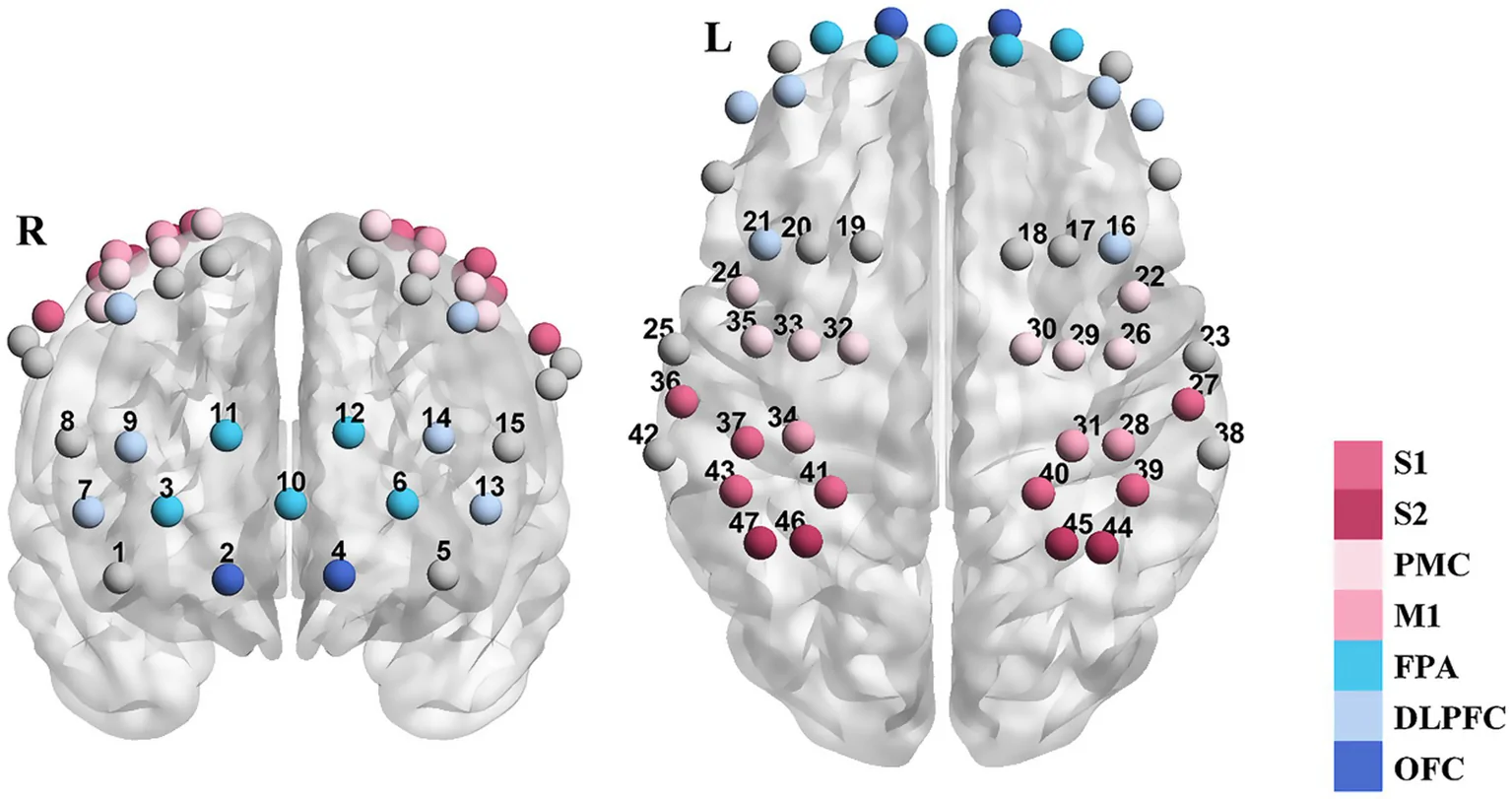
The cortical locations correspond to the channels.

### 2.4 fNIRS data processing

First, the bad channels were removed using the hmrBadChannels function in Homer2 toolkit (default parameters) according to the following criteria: (1) a coefficient of variation of the raw intensity signal exceeding 15% (SD/mean), indicating an unstable or low-quality signal; (2) signal saturation or intensity values falling outside the physiological range; and (Lee et al., 2018) channels with flat traces or abrupt signal drift identified by visual inspection. The remaining raw data were subsequently converted into optical density data. Next, the data were prepared, preprocessed, and statistically analyzed using the MATLAB-based NIRS-KIT (V2.0) toolkit (Hou et al., 2021). To obtain stable signals, we removed data from the first 60 s and last 15 s and then preprocessed the data for detrending, TDDR motion correction, and IIR bandpass filtering 0.01–0.08 Hz. Because oxyhemoglobin (oxy-Hb) concentration changes more directly reflect regional cortical activation than deoxyhemoglobin changes and have been shown to be highly consistent with fMRI blood-oxygenation-level-dependent (BOLD) signal, this study focused on changes in oxy-Hb levels (Huppert et al., 2006; Strangman et al., 2002). At the individual level, ALFF and zALFF, which are commonly used indicators of regional spontaneous brain activity, were calculated in the 0.01–0.08 Hz band. zALFF is the normalized ALFF, which improves the normality of the distribution of individual data by subtracting the global mean of all channels from each channel and then dividing by the standard deviation of all channels, which is more consistent with subsequent normal-based group-level statistics (Zou et al., 2008; Zuo et al., 2010). Therefore, zALFF values were used to characterize differences in brain activity in all group-level statistical analyses.

The rationale for choosing ALFF is as follows. ALFF quantifies the total amplitude of spontaneous low-frequency oscillations (0.01–0.08 Hz) and is a widely used index of regional spontaneous neural activity. Three considerations motivated this choice. First, ALFF is frequency-specific: it isolates spontaneous low-frequency components while excluding low-frequency drift and high-frequency physiological noise (cardiac ~0.6–1 Hz; respiratory ~0.2–0.3 Hz), which simple time-domain metrics (e.g., standard deviation) cannot separate (Hou et al., 2021). Second, ALFF ensures direct comparability with previous EO-EC fMRI studies, which used ALFF to demonstrate opposite EO-EC changes in the frontopolar area and sensorimotor cortex independent of light input (Feng et al., 2021). Third, ALFF is standardized in fNIRS toolkits (Hou et al., 2021; Zou et al., 2008; Zuo et al., 2010) and reliably measurable in both EO and EC resting states (Zou et al., 2015; Liu et al., 2013; Yuan et al., 2018).

### 2.5 Statistical analysis

SPSS software (IBM SPSS version 25) was used to analyze the demographic and clinical data of the two groups. A two-sample *t*-test was used when the data were normally distributed, and the Mann–Whitney *U* test was used when the data were not normally distributed or violated homogeneity of variance. Chi-square (*χ*^2^) analysis was used to analyze sex differences. Based on the MNI coordinates of each channel, we selected 34 channels of interest covering the orbitofrontal cortex (OFC) (channels 2 and 4), frontopolar cortex (FPA) (channels 3, 6, 11, and 12), dorsolateral prefrontal cortex (DLPFC) (channels 7, 9, 13, 14, 16, and 21), premotor cortex (PMC) (channels 22, 24, 26, 29, 30, 32, 33, and 35), primary motor cortex (M1) (channels 28, 31, and 34), somatosensory cortex (S1) (channels 27, 36, 37, 39, 40, 41, 42, and 43), and secondary sensory cortex (S2) (channels 44, 45, 46, and 47).

Two-way repeated-measures analysis of variance (ANOVA) was performed with the condition (EO versus EC) as the within-subject factor and the group (BSP versus HC) as the between-group factor. The degree of freedom (df) and *p-*value were corrected by the Greenhouse–Geisser or Huynh-Feldt method according to the results of the Mauchly sphericality test. The partial *η*^2^ was used for the effect size of the statistical test. All fNIRS data were corrected by the false discovery rate (FDR), and *p* < 0.05 was considered to indicate statistical significance.

## 3 Results

### 3.1 Demographic data

There were no statistically significant differences in age, sex, or years of education between the BSP group and the HC group (*p* > 0.05) (Table 1).

**Table 1:** Demographic and clinical characteristics of the participants.

Characteristics	BSP (*n* = 96)	HC (*n* = 31)	*p*
Age (years)	60.25 ± 10.96	58.16 ± 7.23	0.323
Sex M/F	31/65	11/20	0.743
Education (years)	8.37 ± 3.14	9.35 ± 2.60	0.118
JRS	5.43 ± 0.86	—	—
Duration (months)	52.35 ± 39.49	—	—

BSP, blepharospasm; HC, healthy controls; JRS, Jankovic Rating Scale; −, not applicable.

### 3.2 Brain activity differences

In the HC group, there were significant differences between the EC and EO conditions. The activity of the FPA and OFC was lower, while the activity of the left M1 and bilateral S2 was greater in the EO conditions than in the EC condition (Table 2 and Figure 2).

**Table 2:** Brain regions with significant differences in brain activity between EO and EC in the HC group.

EO vs EC	ROI	Channel	MNI			*T*	*p*
			*x*	*y*	*z*		
EO < EC	FPA	3	30	67	7	−6.89	<0.001
		6	−28	69	9	−4.75	<0.001
	OFC	2	15	72	−9	−5.44	0.000
		4	−12	72	−8	−3.36	0.002
EO > EC	L. M1	34	−34	−26	73	3.30	0.003
	L. S1	41	−27	−39	75	2.72	0.011
	R. S2	44	38	−52	68	3.92	<0.001
	L. S2	46	−33	−51	73	3.47	0.002

R., right; L., left.

**Figure 2:**
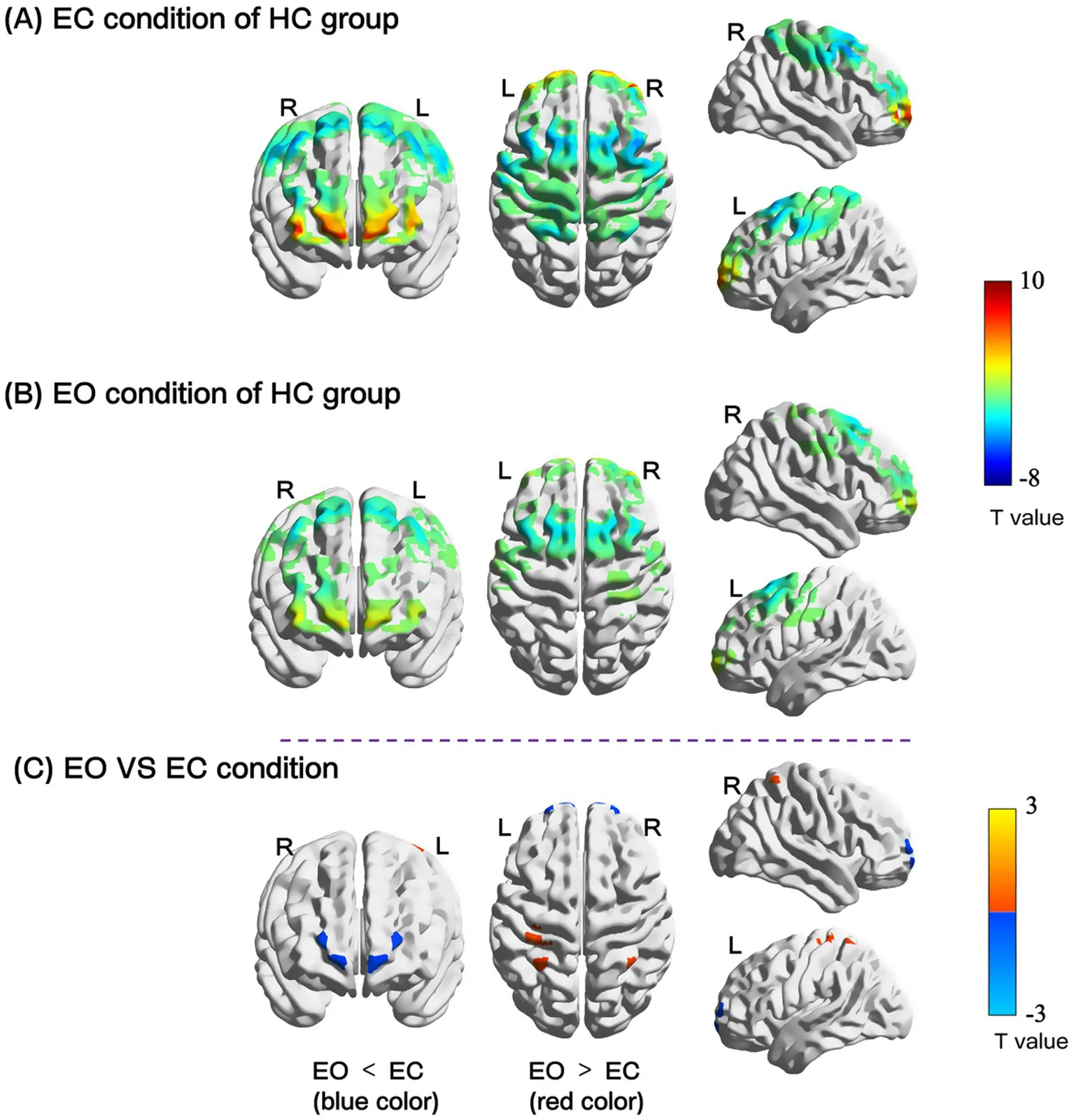
Comparison of cerebral cortex activity between EO and EC state in HC group. **(A)** Brain activity in HC group in the EC condition. **(B)** Brain activity in HC group in the EO condition. **(C)** Brain regions showed significant differences between EO and EC, the activity of the FPA and OFC was lower (blue color), while the activity of the left M1 and bilateral S2 was greater (red color) in the EO conditions than in the EC condition.

In the BSP group, the activity of the FPA and DLPFC was greater, while the activity of the bilateral S1 and right S2 was lower in the EO condition than in the EC condition (Table 3 and Figure 3).

**Table 3:** Brain regions with significant differences in brain activity between EO and EC in the BSP group.

EO vs EC	ROI	Channel	MNI			*T*	*p*
			*x*	*y*	*z*		
EO > EC	FPA	3	30	67	7	3.75	<0.001
		10	−0.4	68	9	2.59	0.01
		11	15	66	26	3.26	0.002
		12	−14	66	26	3.28	0.002
	L. DLPFC	14	−36	56	25	2.78	0.007
	R. DLPFC	7	49	51	7	2.69	0.008
		9	39	56	23	4.56	<0.001
EO < EC	R. S1	27	59	−18	54	−4.28	<0.001
		39	45	−39	64	−3.11	0.003
		40	23	−40	77	−3.26	0.002
	L. S1	43	−49	−39	61	−3.736	0.000
	R. S2	44	38	−52	68	−2.713	0.008

**Figure 3:**
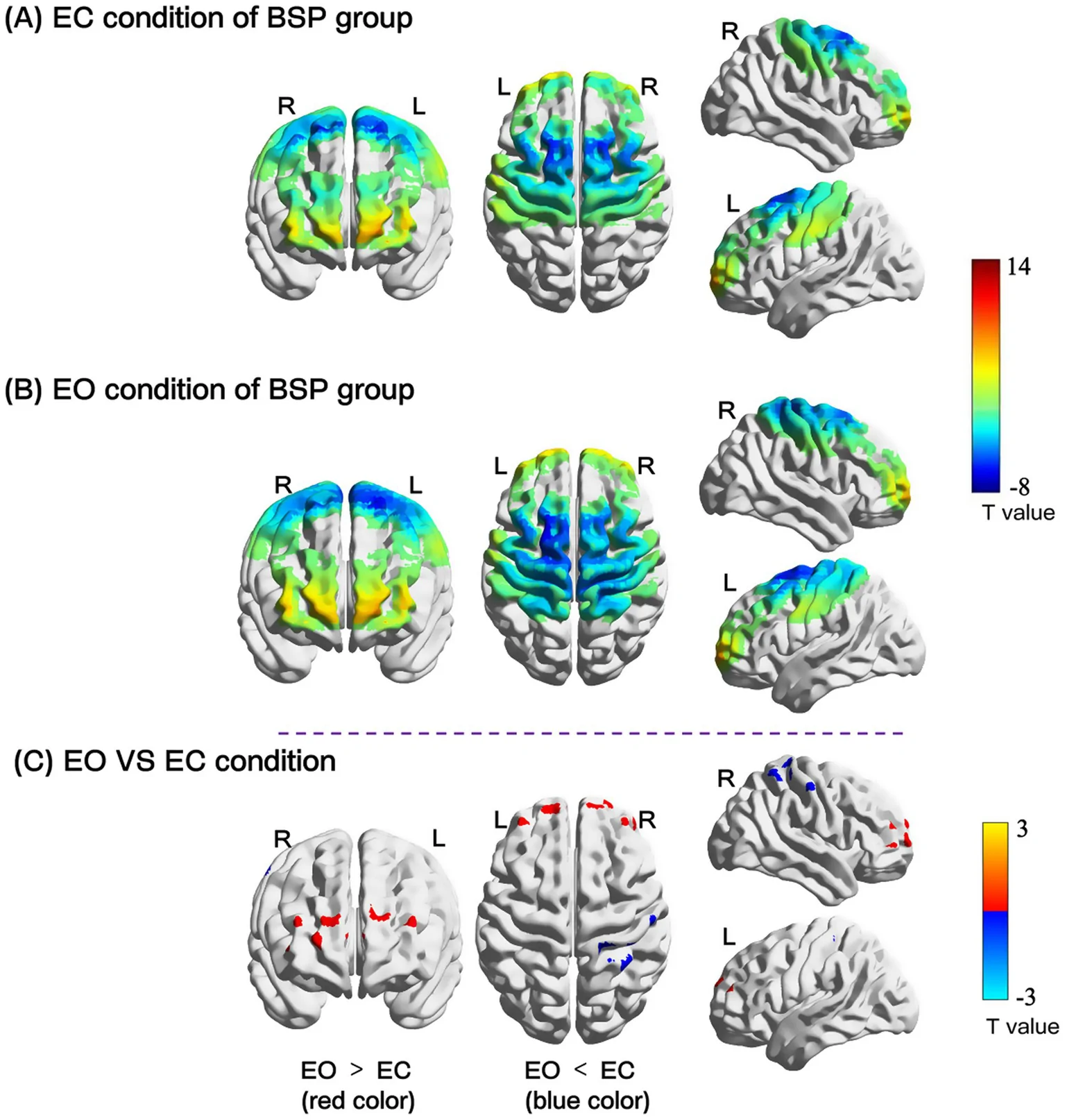
Comparison of cerebral cortex activity between EO and EC in BSP group. **(A)** Brain activity in BSP group in the EC condition. **(B)** Brain activity in BSP group in the EO condition. **(C)** Brain regions showed significant differences between EO and EC, the activity of the FPA and DLPFC was greater, while the activity of the bilateral S1 and right S2 was lower in the EO conditions than in the EC condition.

In the EC condition, activity in the OFC and FPA was decreased in the BSP group compared with the HC group. In the EO condition, the activity of the FPA and DLPFC increased, while the activity of the right PMC, bilateral M1, bilateral S1 and bilateral S2 decreased in the BSP group compared with the HC group. A two-sample *t-*test (Table 4 and Figure 4) was used for the main effect results, and repeated-measures ANOVA was used for the interaction effect.

**Table 4:** Comparison of brain activity between BSP group and HC group.

Condition	BSP vs HC	ROI	Channel	MNI			*T*	*p*
				*x*	*y*	*z*		
EC	BSP < HC	OFC	2	15	72	−9	−3.67	<0.001	FPA	3	30	67	7	−3.99	<0.001
		EO	BSP > HC	R. FPA	3	30	67	7	2.37	0.019	L. FPA	6	−28	69	9	3.02	0.003	R. DLPFC	9	39	56	23	2.49	0.012		16	−36	56	25	2.55	0.014	L. DLPFC	14	41	19	57	2.38	0.019	BSP < HC	R. PMC	30	42	−28	69	−2.47	0.015	R. M1	28	20	−5	77	−2.99	0.001		31	31	−28	74	−3.33	0.003	L. M1	34	−34	−26	73	−3.10	0.002	L. S1	37	−46	−27	68	−3.35	0.001		41	−27	−39	75	−2.51	0.014	R. S1	39	45	−39	64	−2.46	0.015		40	23	−40	77	−3.52	<0.001	R. S2	45	28	−52	73	−2.42	0.017	L. S2	46	−33	−51	73	−2.58	0.011

R., right; L., left.

**Figure 4:**
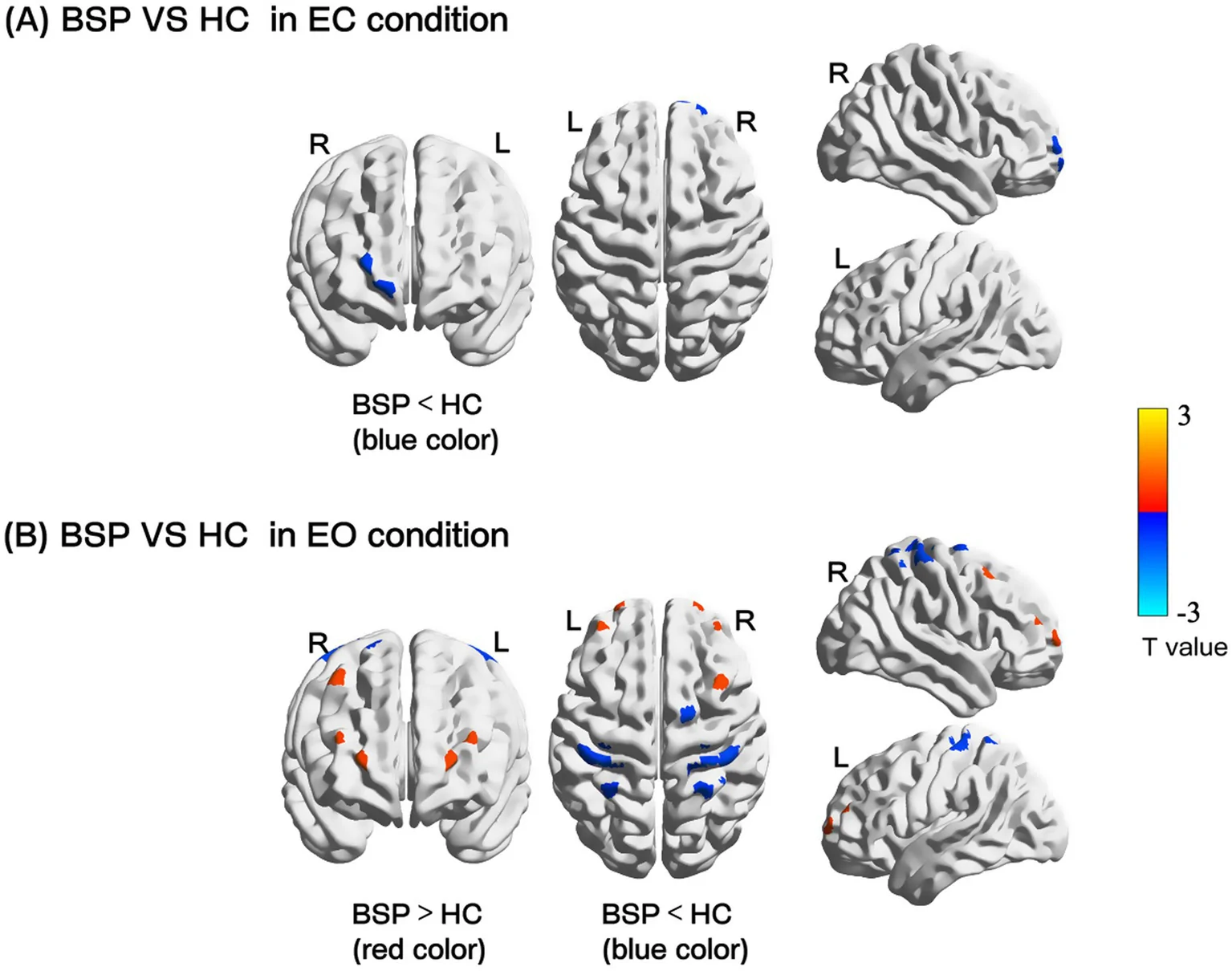
Comparison of brain activity between BSP group and HC group. **(A)** Brain regions showed significant differences between BSP and HC groups in the EC condition. **(B)** Brain regions showed significant differences between BSP and HC groups in the EO condition.

### 3.3 Receiver operating characteristic (ROC) curve analysis

The zALFF values of the right S2 (channel 44) and right FPA (channel 3) in the BSP group showed EO-EC patterns opposite to those in the HC group (Tables 2, 3). This observation directly matched our *a priori* hypothesis that BSP involves an imbalanced shift between the prefrontal and sensorimotor cortices across the EO and EC states; accordingly, we defined the difference in zALFF between channel 44 and channel 3 (ch44-ch3) and calculated EO (ch44-ch3) minus EC (ch44-ch3) as “delta EO-EC” index for both groups (Table 5, Figure 5). Channel 44 and 3 were selected because, among the 34 channels of interest, they form the only pair exhibiting significant EO-EC differences with opposite directions in the two groups (HCs: ch3 EO < EC, *T* = −6.89, *p* < 0.001; ch44 EO > EC, *T* = 3.92, *p* < 0.001, Table 2; BSP: ch3 EO > EC, *T* = 3.75, *p* < 0.001; ch44 EO < EC, *T* = −2.71, *p* = 0.008, Table 3). Although other channels showed broadly similar group differences, none showed this complete cross-over in both groups. We emphasize that the index was derived from a physiologically motivated hypothesis rather than from a data-driven feature selection procedure; nevertheless, because the index and the ROC evaluation were derived from the same cohort, the results should be interpreted as discovery-stage evidence. The receiver operating characteristic (ROC) analysis showed that delta EO-EC discriminated BSP patients from HCs with an area under the curve (AUC) = 0.92, a sensitivity of 90.32%, specificity of 80.65%, Youden index of 0.797, and a corresponding cutoff of 0.616 (Figure 5); these estimates require confirmation in an independent cohort (see Section 4.4).

**Table 5:** zALFF value difference between right S2 (Ch44) and FPA (Ch3).

Condition	BSP	HC	*p*
EO	−0.67 ± 1.32	0.80 ± 1.50	0.009
EC	−0.19 ± 1.22	−1.32 ± 1.16	<0.0001
Delta EO-EC difference	−0.48 ± 0.98	1.40 ± 1.04	<0.0001

**Figure 5:**
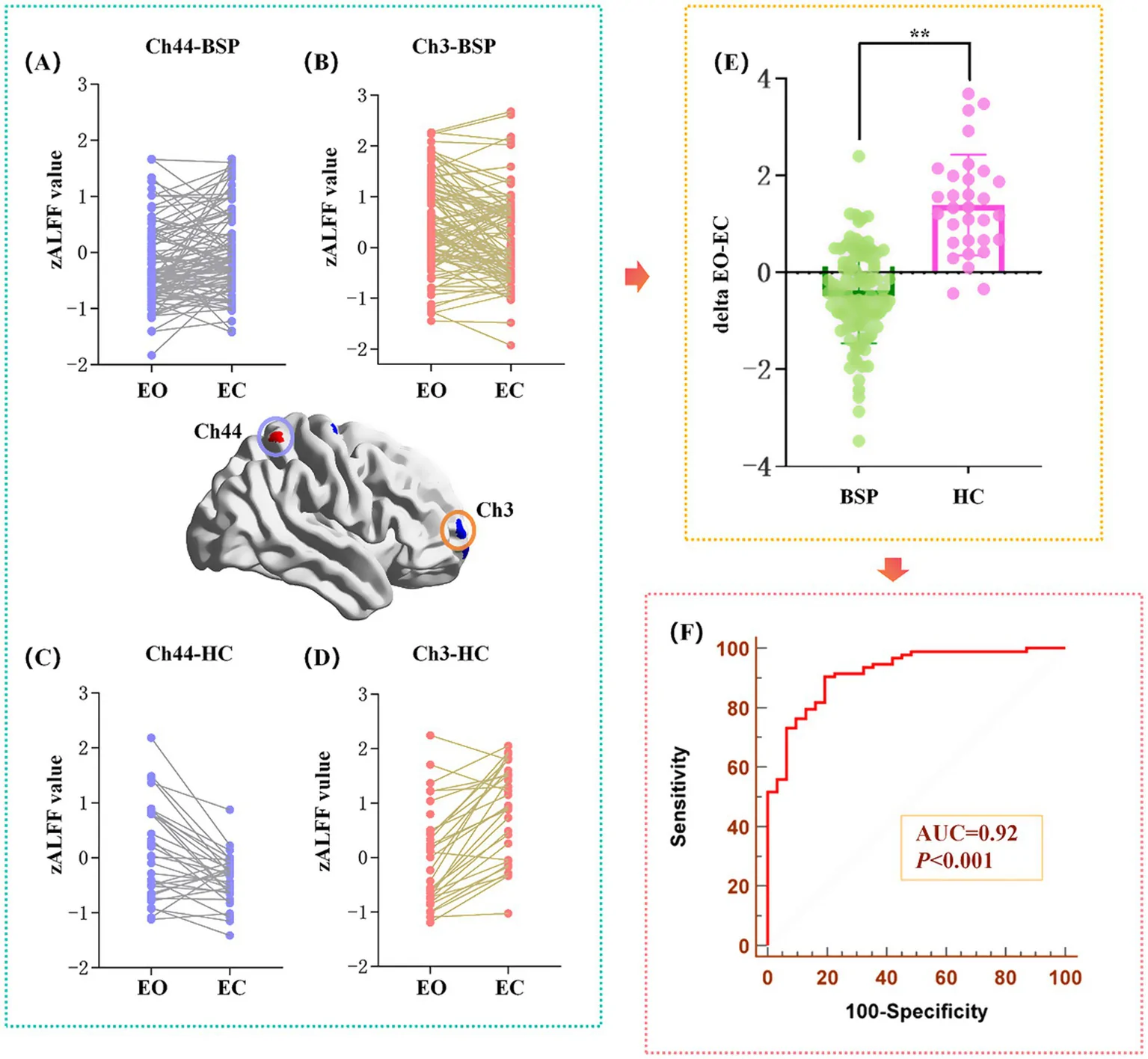
The difference in delta EO-EC between the BSP and HC groups and ROC curve analysis **(A)** zALFF value of channel 44 in BSP group. **(B)** zALFF value of channel 3 in BSP group. **(C)** zALFF value of channel 44 in HC group. **(D)** zALFF value of channel 3 in HC group. **(E)** delta EO-EC in the BSP and HC groups. **(F)** ROC curve of delta EO-EC for distinguishing BSP patients from HCs.

## 4 Discussion

Previous functional brain imaging studies of BSP patients have been performed mainly in the resting state with eyes closed, and regions involved in abnormal brain function activity during the spasm period cannot be directly observed. The present study revealed that the BSP and HC groups exhibited significantly different brain activity patterns in the EC condition, EO condition, and delta EO-EC. In the EC condition, the OFC and FPA showed decreased activity in BSP patients compared with that in the HCs. In the EO condition, compared with those in the HCs, the cerebral cortex activity in the FPA and DLPFC in the BSP group increased, while the activity in the right PMC, bilateral M1, bilateral S1 and bilateral S2 decreased. Interestingly, we found increased activity in the sensorimotor cortex and decreased activity in the prefrontal cortex in the HC group in the EO condition compared with the EC condition; however, in contrast, sensorimotor cortex activity was decreased in the BSP group under EO conditions, while prefrontal brain activity was increased. Therefore, we performed further analyses and found that the delta EO-EC difference showed promising discriminative accuracy as a candidate indicator for BSP in this discovery cohort.

### 4.1 Mechanism of abnormal brain activity in BSP under EC conditions

In this study, we found that brain activity in the FPA and OFC in the prefrontal cortex was lower in BSP patients than in HCs under the EC condition. These findings are consistent with those of previous studies (Mesbah et al., 2023; Bugalho et al., 2008; Vilany et al., 2017; Tomić et al., 2023). The FPA (Brodmann area 10) and OFC are the two most anterior prefrontal regions and are involved in high-level cognitive and emotional regulation, with extensive fiber connections to the adjacent DLPFC, OFC, and higher-order association cortices (Christoff et al., 2001; Ramnani and Ramnani, 2004). The FPA and OFC exhibit a large number of projections to the basal ganglia, including the putamen and caudate nucleus, and regulate the function of the striatum (Averbeck et al., 2014; van Holstein et al., 2018). FPA lesions are associated with impaired performance in abstract reasoning, problem solving or multitasking, and emotional and behavioral control (Roca et al., 2010; Bramson et al., 2023). BSP patients have been confirmed to have anxiety, depression and mild cognitive impairment (Defazio et al., 2017; Defazio et al., 2022). Within this framework, reduced prefrontal ALFF in BSP patients at rest (EC condition) suggests weakened tonic top-down modulation of the sensorimotor cortex, which may contribute to the failure to suppress involuntary orbicularis oculi activity-a mechanism consistent with the reduced cortical inhibition reported in BSP (Hao et al., 2023; Perruchoud et al., 2014) and may also relate to the disorder’s high psychiatric comorbidity burden (Defazio et al., 2017; Defazio et al., 2022).

Abnormal OFC activation has also been reported previously (Yang et al., 2013). Given that OFC dysfunction can disrupt “top-down” regulation from higher-order prefrontal regions over motor execution (Burgmer et al., 2006; Roelofs et al., 2006; Cojan et al., 2009; Voon et al., 2011), the reduced OFC activity observed here may further contribute to weakened prefrontal control of the sensorimotor loop, and is consistent with the insufficient OFC recruitment reported in BSP patients during cognitive tasks (Bramson et al., 2023).

Previous studies have shown that PFA activity is lower in healthy subjects with eyes closed than in those with eyes open, which is inconsistent with the results of this study. One possible, though untested, explanation is that the analyses in the previous studies were all performed in the supine resting state, whereas the subjects in this study maintained a seated position; postural demand is known to modulate prefrontal and sensorimotor activity (Teo et al., 2018; Mihara et al., 2008). Further studies directly comparing seated and supine positions within the same cohort are needed to verify this account.

### 4.2 Mechanisms of abnormal brain activity in BSP under EO conditions

To further understand the brain mechanisms associated with spasm occurrence in the real world, we explored the activation/impairment representation of brain function during the spasm period under EO conditions. In this study, healthy controls had no difficulty with spontaneous blinking during EO, while BSP patients inevitably attempted to control eyelid opening or suppress involuntary eye closure due to difficulty in opening eyes and involuntary eye closure. Moreover, we observed that BSP patients exhibited increased activity in the bilateral PFC and decreased activity in the sensorimotor cortex. Combined with the findings of previous studies, these findings suggest that insufficient activation of the sensorimotor cortex may lead to insufficient cortical inhibition and induce blepharospasm, while increased activity in the PFC may be a recruitment mechanism for the regulation of higher cognitive function.

Several previous studies have reported impaired function of the sensorimotor cortex in patients with BSP. Our previous work also showed decreased sensorimotor network functional connectivity in BSP patients (Huang et al., 2017; Huang et al., 2023). VBM studies have shown increased gray matter density in the bilateral primary sensorimotor cortex, which is positively correlated with the duration and severity of the disease, while functional connectivity and white matter fiber connectivity are significantly reduced (Suzuki et al., 2011). Therefore, it is believed that the increased gray matter density in the sensorimotor cortex may be a secondary effect caused by long-term overactivity or a compensatory response to inefficiency/insufficiency rather than a predisposing factor (Zou et al., 2008; Battistella et al., 2017). Sensory disturbance is an important clinical symptom of BSP, and patients often experience pain, discomfort, an impaired sensory discrimination threshold, and impaired sensory gating (Defazio et al., 2017; Hao et al., 2023; Huang et al., 2015; Huang et al., 2023). A number of studies have confirmed abnormal brain activity and functional connectivity in the S1 of BSP patients (Huang et al., 2017; Luo et al., 2022; Nguyen et al., 2020; Pan et al., 2021). Magnetic resonance diffusion tensor tractography also showed abnormal nodal efficiency in the S1 (Guo et al., 2021). Sensory disturbances are considered an inherent part of the pathophysiology of blepharospasm because they often precede clinical symptoms, and insufficient activation of S1 and S2 is found in both affected and unaffected body parts (Conte et al., 2017; Dresel et al., 2011).

The PMC is responsible for the initiation of voluntary movement and may be related to the initiation of eyelid opening (Wolpert et al., 1995; Chouinard and Paus, 2006). Several previous fMRI studies have identified a loss of function in the key regions of information transmission (hubs) of the PMC in BSP patients (Ramdhani et al., 2014). Diffusion tractography also revealed reduced connectivity between the right PMC and left occipital thalamus, which is an important relay station between the visual and sensorimotor networks (Mantel et al., 2022). The sensorimotor integration model involves the coordination of high-level and low-level nodes, and the PMC is at a high level in the sensorimotor circuit, where it modulates signals from low-level nodes and projects them back to the S1 to calibrate movements and execute commands with other high-level nodes (Perruchoud et al., 2014; Wolpert et al., 1995). Compared with the static balance standing task, the PMC and supplementary motor areas were activated in the dynamic balance task but not in the motor imagery task (Almulla et al., 2022). Therefore, the PMC may be more important in the actual execution of movement. In addition, reduced brain connectivity in the PMC may lead to impaired peripheral inhibition, thus causing excessive muscle contractions and involuntary movements (Pan et al., 2021; Smitha et al., 2017). Therefore, dysfunction of the PMC may lead to difficulty in eyelid opening and in inhibiting involuntary eyelid closure.

The activity of the FPA and DLPFC in BSP patients increased significantly in the EO condition. The PFC supports higher cognitive functions and projects extensively to other cortical, subcortical, brainstem and cerebellar regions. Greater PFC activation may help improve attention and contribute to more accurate feedforward postural control (Bunce and Barbas, 2011; Herrmann et al., 2008). It has been suggested that this increase in PFC activity is intended to compensate for the degradation of the sensorimotor system. There is a wide range of mutual afferent and efferent fibers between the FPA and DLPFC, among which there are more afferent fibers from the FPA to the DLPFC, covering almost the whole DLPFC (Petrides and Pandya, 2007). The DLPFC is a core node involved in the selective allocation of attentional resources, and its activation may be related to the attentional process of postural balance control (Luks et al., 2007). Studies in which patients maintained a sitting posture showed a gradual increase in DLPFC activation with increasing complexity of sensory afferent information (Yang et al., 2013; Mihara et al., 2008). Increasing complexity in the balance task leads to a more significant level of hemodynamic response in the PFC (Smitha et al., 2017; Verghese et al., 2017). Since the peak age of BSP onset is in the 60s, its pathogenesis is likely related to a decrease in postural motor balance control function related to brain aging. The postural balance control task revealed that elderly individuals had greater DLPFC activation than young individuals but lost the lateralization characteristic of young individuals, suggesting that increased DLPFC activation may be involved in brain aging (Teo et al., 2018). When young subjects performed the dual task of cognition and postural balance, the intensity of prefrontal activity increased with the difficulty of the task. However, in elderly subjects, when the task difficulty exceeded a certain level, PFC activity showed instability (Mihara et al., 2008). Increased PFC activity is a predictor of the risk of falls in elderly individuals when they are performing walking tasks (St George et al., 2021). It is well established that activity in the PFC increases as the brain ages. There are two theoretical explanations for this increase in PFC activity. The earlier and seemingly more accepted hypothesis is the “compensation hypothesis,” which proposes that when posterior cortical function is damaged or degenerated, a compensatory function shifts from posterior to anterior (PASA) to maintain cognitive performance (Reuter-Lorenz et al., 2008; Grady, 2012). This compensation is defined as neural recruitment to enhance task performance (McDonough and Madan, 2021). An alternative hypothesis is that increased prefrontal activity may instead be a manifestation of decreased efficiency or specificity due to the structural and neurochemical impairments that accompany aging (West, 1996; Nyberg et al., 2012; Morcom and Henson, 2018). Increased prefrontal activity has also been reported in studies of sensorimotor integration dysfunction and postural regulation dysfunction in individuals with other types of movement disorders, suggesting that increased prefrontal activity also plays an important role in sensorimotor regulation (Morcom and Henson, 2018). The spasms of BSP patients can be alleviated or even relieved when they are lying down, relaxing or performing skilled tasks, but they are significantly aggravated when walking or feeling stressed or angry. This may represent a recruitment overload of the PFC to coordinate the eye-opening state and the walking/emotional dual task. Stopping other tasks or using sensory tricks to compensate for afferent information may reduce PFC overloading. Therefore, bilateral prefrontal overactivation in the BSP may also be a manifestation of brain aging, but whether the specific mechanism involved was PASA or whether this was due to reduced efficiency/specificity remains to be further investigated.

In the EO condition, the BSP patients had to perform a dual task of maintaining balance in the sitting position and opening their eyelids. Therefore, it is believed that the decreased activation of the sensorimotor cortex and insufficient inhibitory regulation of the downstream nodes produce spasticity symptoms, and overactivation in the FPA and DLPFC is a compensatory response that occurs to maintain dual-task performance to regulate decreased activation in the sensorimotor cortex.

Taken together, the opposite ALFF patterns between groups can be interpreted as a reversed “spontaneous activity-inhibition balance” in the two cortical regions. In HCs, the EC condition favors an interoceptive, internal state: greater prefrontal ALFF (FPA/OFC) reflects enhanced internal cognitive processing, whereas lower sensorimotor ALFF reflects reduced somatosensory afferent processing. In BSP patients, this pattern is inverted: greater sensorimotor ALFF during EC suggests abnormal spontaneous activity or insufficient inhibition in the sensorimotor cortex even in the symptom-silent state, whereas lower prefrontal ALFF suggests weakened top-down modulation of the sensorimotor loop at rest. During EO, the persistence of reduced sensorimotor ALFF alongside exaggerated prefrontal ALFF in BSP suggests that eye opening exposes the insufficient sensorimotor inhibition underlying difficulty in maintaining eye opening, and simultaneously triggers compensatory over-recruitment of the prefrontal cortex. Thus, the group differences in ALFF are best understood not as isolated regional abnormalities but as opposite regional shifts of a prefrontal-sensorimotor regulatory imbalance, which also underlies the rationale for the delta EO-EC index.

### 4.3 Delta EO-EC differences as a candidate biomarker of BSP

The difference in delta EO-EC between the BSP and HC groups showed an opposite pattern, and the difference in the right S2 and right FPA showed promising discriminatory value for distinguishing BSP patients from HCs, which was an intriguing finding in this discovery cohort. As described in Section 3.3, channels 44 and 3 were selected because they form the only channel pair showing a complete EO-EC cross-over between groups, consistent with the prefrontal-sensorimotor imbalance framework discussed above. Previous studies have confirmed that there are stable differential representations of the topological and dynamic properties of the primary sensorimotor cortex, prefrontal cortex, and visual cortex activity and brain functional networks between EO and EC conditions in HCs (Zou et al., 2015; Liu et al., 2013; Yuan et al., 2018; Xu et al., 2014; Weng et al., 2020). The EO condition is a fundamental behavior that directs attention to the external world, which shifts our brain from an internal to an external state; therefore, EO-EC condition switching may be a switch between interoceptive and exteroceptive states (Feng et al., 2021; Agcaoglu et al., 2019). fMRI studies have shown that EO/EC conditions mainly mediate on/off switches that influence cerebral activation patterns rather than light signal afferents (Feng et al., 2021; Agcaoglu et al., 2019). Moreover, ALFF analysis via fMRI revealed that brain activity in the frontopolar area and sensorimotor cortex exhibited opposite changes in EO and EC conditions, and these changes were not related to light or visual deprivation, indicating that these changes are endogenous natural effects related to EO and EC conditions (Feng et al., 2021). However, most of the previous studies on EO-EC differences were performed in the supine resting state, whereas this study observed EO-EC differences in BSP patients in the sitting posture. It is plausible that a sitting position is closer to a real-world state than lying flat and may therefore have better ecological validity; however, the present design did not directly compare postures, and this interpretation remains to be confirmed by future studies manipulating posture within the same cohort. Notably, in the present seated posture, HCs showed greater sensorimotor and lower prefrontal activity in EO than in EC, directionally consistent with previous supine studies (Yuan et al., 2018; Agcaoglu et al., 2019), whereas BSP patients showed the opposite pattern.

In the HC group, greater activation in the PFC in the EC condition may be due to the greater reliance on internal information to maintain the sitting posture, while the increased external information input in the EO condition leads to increased activation in the sensorimotor cortex and decreased activation in the PFC. However, in the BSP group, insufficient activation of the sensorimotor cortex during eye opening led to difficulty in opening the eyelid and maintaining eye opening, requiring greater recruitment of the prefrontal cortex to regulate eye opening behavior through increased attention allocation and internal cognitive resource regulation.

Interestingly, we found that the difference in delta EO-EC between the right S2 region and the right FPA region showed promising discrimination value for identifying BSP patients in this discovery cohort and may serve as a candidate biomarker for the differential diagnosis of BSP. Because the index and the ROC evaluation were derived from the same cohort, the reported performance may be optimistically biased and should be interpreted as preliminary evidence pending external validation in an independent cohort. If confirmed in an independent cohort, this simple, rapid metric may aid early diagnosis of BSP and reduce diagnostic delay.

### 4.4 Limitations of the current study

There are still several limitations in this study. First, this study did not compare the correlations among disease duration, severity of illness and brain function; second, this study did not consider the impact of nonmotor symptoms; and third, this study was cross-sectional and could not identify behavioral or brain mechanism changes in patients over time. Fourth, the ROC analysis was performed in the same cohort from which the delta EO-EC index was derived; although this index was defined on the basis of an *a priori* hypothesis rather than a data-driven feature search, the reported AUC may still be optimistically biased, and external validation in an independent cohort is required before this metric can be considered a validated diagnostic biomarker for BSP. Fifth, although the EO paradigm was standardized (fixation on a marker at 1 meter without deliberate eyelid control) and subjects with excessive eye closure (>30 s) were excluded, subtle differences in task demands, compensatory effort, attention, and visual processing between the BSP and HC groups cannot be entirely excluded. Future studies using parametric manipulation of visual load or sensory-trick paradigms are needed to further disentangle spasm-related mechanisms from compensatory processes. Sixth, the discrepancies between our findings and previous supine-position studies were attributed to the seated posture, but posture was not directly manipulated in the present design, and this explanation remains speculative; studies comparing seated and supine positions within the same cohort are needed. Seventh, although subjects with cumulative eye-closure exceeding 30 s were excluded and all included participants completed the EO acquisition, episode-level ocular spasms during EO were not prospectively quantified; future studies combining fNIRS with synchronized video-based spasm scoring are needed to further validate the EO comparison in the BSP group.

Despite these limitations, this study provided further evidence for the ability of fNIRS to detect differences in the patterns of EC-EO in patients with BSP. These results could aid future biomarker studies and further provide guidance and support for the clinical diagnosis of BSP.

## Data Availability

The raw data supporting the conclusions of this article will be made available by the authors, without undue reservation.
